# Signatures of local adaptation in the spatial genetic structure of the ascidian *Pyura chilensis* along the southeast Pacific coast

**DOI:** 10.1038/s41598-020-70798-1

**Published:** 2020-08-24

**Authors:** Nicolás I. Segovia, Claudio A. González-Wevar, Pilar A. Haye

**Affiliations:** 1 Departamento de Biología Marina, Facultad de Ciencias del Mar, Universidad Católica del Norte, Coquimbo, Chile; 2 Departamento de Ciencias Ecológicas, Facultad de Ciencias, Instituto de Ecología Y Biodiversidad IEB, Universidad de Chile, Santiago, Chile; 3 Instituto de Ciencias Marinas Y Limnológicas (ICML), Facultad de Ciencias, Universidad Austral de Chile, Valdivia, Chile; 4 Centro FONDAP de Investigaciones en Dinámica de Ecosistemas de Altas Latitudes (IDEAL), Universidad Austral de Chile, Valdivia, Chile

**Keywords:** Evolution, Ecology

## Abstract

The highly heterogeneous Humboldt Current System (HCS) and the 30°S transition zone on the southeast Pacific coast, represent an ideal scenario to test the influence of the environment on the spatial genomic structure in marine near-shore benthic organisms. In this study, we used seascape genomic tools to evaluate the genetic structure of the commercially important ascidian *Pyura chilensis,* a species that exhibits a low larval transport potential but high anthropogenic dispersal. A recent study in this species recorded significant genetic differentiation across a transition zone around 30°S in putatively adaptive SNPs, but not in neutral ones, suggesting an important role of environmental heterogeneity in driving genetic structure. Here, we aim to understand genomic-oceanographic associations in *P. chilensis* along the Southeastern Pacific coast using two combined seascape genomic approaches. Using 149 individuals from five locations along the HCS, a total of 2,902 SNPs were obtained by Genotyping-By-Sequencing, of which 29–585 were putatively adaptive loci, depending on the method used for detection. In adaptive loci, spatial genetic structure was better correlated with environmental differences along the study area (mainly to Sea Surface Temperature, upwelling-associated variables and productivity) than to the geographic distance between sites. Additionally, results consistently showed the presence of two groups, located north and south of 30°S, which suggest that local adaptation processes seem to allow the maintenance of genomic differentiation and the spatial genomic structure of the species across the 30°S biogeographic transition zone of the Humboldt Current System, overriding the homogenizing effects of gene flow.

## Introduction

Most marine taxa, and especially near-shore invertebrates, have free-living larval stages allowing dispersal across heterogeneous environments that lack evident barriers to gene flow^1–3^. Dispersal has been often assumed to be an effective mechanism of maintaining connectivity between local populations^4,5^. Nonetheless, many species display inconsistent patterns of genetic structure with those predicted from their developmental modes^6–9^. For instance, several direct developers are broadly distributed over thousands of kilometers, probably through long-distance dispersal mediated by rafting^6,8,10–13^, while many broadcast-spawners exhibit high levels of genetic structure at narrower geographical scales^2,7,14^. Currently, it is widely accepted that evolutionary forces, despite dispersal, can shape the genetic structure of a species across its distribution. Such factors include natural selection, phenotype-environmental mismatch and local adaptation, which may override the genetic signatures expected solely on the basis of gene flow^15–17^.

Understanding how environmental factors influence the contemporary spatial distribution of genomic diversity has become a main goal in evolutionary ecology and biogeography^18–23^. Many relevant environmental variables are heterogeneous across a species’ distributional range and generate differential local selective pressures that may lead to major differences in global patterns of genetic differentiation. Thus, local adaptation can lead to genetic structure across a species’ range even in the presence of high levels of gene flow^5,24,25^.

Landscape genomics combines population genomics and landscape ecology to further understand how biotic and abiotic factors affect the processes associated with local adaptation and population structure^18^. A main focus is the study of adaptive genetic structure associated with environmental data, which has been increasingly applied using Next Generation Sequencing (NGS) techniques through the identification of thousands of polymorphic loci scattered across the genomes^26,27^. However, very few studies on marine benthic invertebrates have attempted to identify loci that are putatively under selection in association with seascape; most studies have use traditional DNA markers^28–33^, and seascape genetic studies have evaluated neutral processes associated with genetic connectivity, niche modeling, as well as the role of physical factors^20,28,33^. Genomic-based studies considering the potential effects of environmental factors in neutral and adaptive genetic variation in marine invertebrates are still scarce^27,32,34–37^.

### The Humboldt current system

The Humboldt Current System (HCS) is an environmentally heterogeneous system and accordingly, a very interesting area to evaluate adaptive genetic structure in marine organisms. This system does not have evident geographic or physical barriers to gene flow along its ~ 2,600 km of linear coastline. Nevertheless, several studies have recorded significant genetic differences between local populations in the area^3,14,38^. Such patterns of genetic structure have mainly been associated with a widely reported biogeographical/phylogeographic transition zone at ~ 30°S^3,39,40^. This transition zone has been attributed to historical factors because only species with low dispersal capacity exhibit signals of marked genetic differentiation^3^. According to Lara et al.^41^, patterns of differentiation in poorly dispersing taxa are probably maintained through environmental differences north and south of 30°S. Therefore, this environmental discontinuity may also have contemporary influence on the genetic structure in species with low dispersal potential along the HCS. Conversely, in species with high dispersive potential, the signals of genetic differentiation across this transition zone have been completely erased by neutral processes probably associated with the homogenizing effect of gene flow^3^.

The ascidian *Pyura chilensis* Molina 1782 is a benthic intertidal tunicate that is endemic to the southeastern Pacific coast, and represents one of the most conspicuous inhabitants of the HCS^42^. Like many other tunicates, *P. chilensis* is considered a poor disperser because it has a very short free-living lecithotrophic larval stage (12–24 h), after which it settles on hard substrate or on the matrix of conspecifics^43–45^. Phylogeographic studies using allozymes^46^, and nucleic acids^8^ suggest low or even a complete lack of genetic structure across the 30°S transition zone of the HCS. The pattern of genetic homogeneity in *P. chilensis* contrasts with those recorded in other near-shore species with low dispersive potential along the study area^3,14,39,40^. The absence of genetic differentiation in *P. chilensis* has been explained by anthropogenic transport promoting high levels of connectivity^8,47^, as recorded in other ascidians worldwide^48,49^. Even though the mechanism associated with the anthropogenic dispersal of *P. chilensis* has not been explicitly evaluated, this tunicate has repeatedly been reported on artisanal boat hulls, buoys and marine installations as an important component of the biofouling communities^50,51^*.* Hence, it is likely that human activities may play an important role in maintaining genetic homogeneity recorded in the species across the HCS. More recently Segovia et al.^47^ using Single Nucleotide Polymorphisms generated with Genotyping-By-Sequencing (SNP-GBS) evaluated neutral and putatively adaptive genetic structure in the species and recorded contrasting results to those previously detected, particularly across the 30°S transition zone. Consistent with traditional markers^8,46^, neutral SNPs loci^47^ showed absence of genetic structure across the 30° zone. In this neutral loci, 30°S structure appear only forcing a sub-optimal clustering. In contrast, the analyses of putatively selective SNP loci showed strong evidence of genomic structure across the transition zone^47^. Such results indicate that marine environmental stressors have a selective role shaping patterns of genomic structure along the HCS and override the effect of gene flow that is most likely mediated by anthropogenic transport^47^.

The aim of this study was to characterize both, neutral and putatively adaptive genomic structure in *P. chilensis* across the 30°S transition zone of the HCS through the combined use of two seascape genomic-oceanographic approaches. Through this, it will be possible gain a better understanding of the role of environmental differences in the spatial genomic structure in a species with low intrinsic dispersion potential but high physical transport along the study area.

## Results

### Genomic data collection

**Figure 1:**
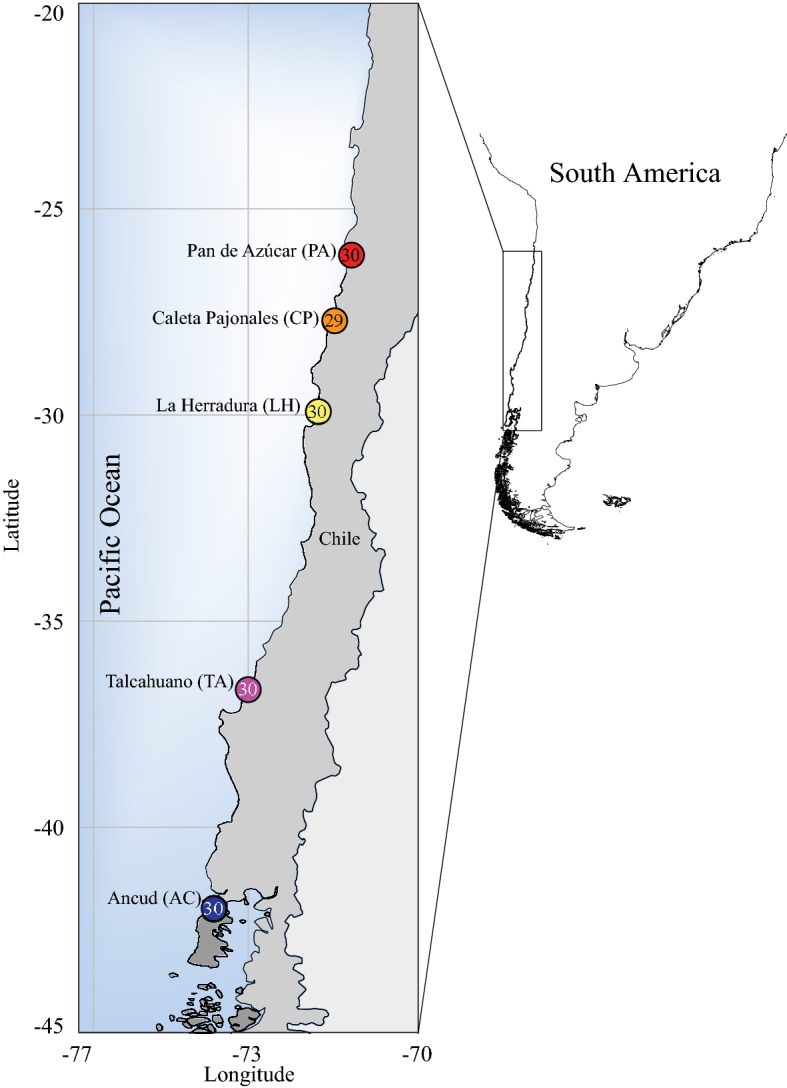
Map of the sampling sites of *P. chilensis* across the Humboldt current System. Numbers correspond to the total number of analyzed individuals per site.

**Figure 2:**
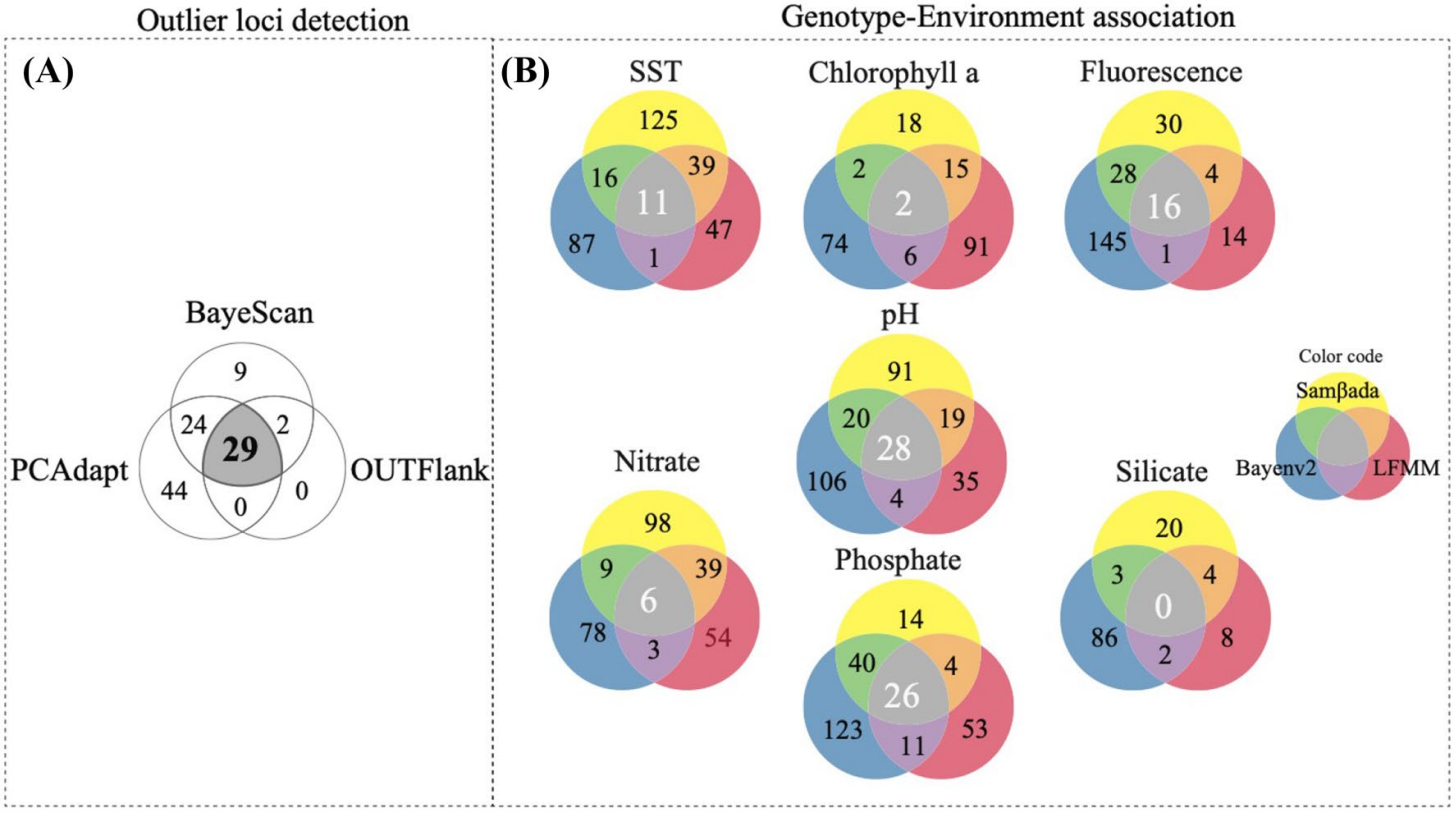
Venn diagrams of the number of putatively adaptive loci detected for *Pyura chilensis *using (**A**) outlier detection approaches and (**B**) genotype-environment associations. Diagram shows all candidate loci detected by Bayescan, Outflank and PCAdapt using genetic differentiation for *outlier* detection and LFMM, Bayenv2 and SAMβADA using oceanographic variables. Figure indicates the exclusives loci detected of each method and the common ones between two and three methods.

A total of 344,964,130 reads with a mean quality score (QF) of 34.34 were obtained from 149 individuals collected from five localities along the southern Chilean coast from two consecutive years (Fig. 1; Table 1). Out of them, 2,024,910 *tags* were successfully retained yielding, before filtering, 102,816 SNPs in the variant call process using TASSEL. Using restrictive bioinformatic filters described by Segovia et al.^46^ for *P. chilensis* (minimum call rate of 85% and Minor Allele Frequency of 0.04), a total of 2,902 SNPs were genotyped. Among these, 67 candidate loci for positive selection and 2,521 as putatively neutral loci were identified using Bayescan analysis. A total of 51 loci (76%) showed strong or very strong evidence of positive selection following the Bayes Factor. Similarly, Out-flank and PCAdapt detected 31 and 97 candidate loci for positive selection, respectively. In consensus, the three different methods detected in common a total of 29 candidate loci for positive selection (Fig. 2A). These loci were used to analyze the putatively adaptive genomic structure of *P. chilensis* based solely in population differentiation analysis (i.e. *F*_ST_-like, ordination) across the HCS transition zone. At the same time, we considered those SNPs explicitly defined as putatively neutral loci (N = 2,521) by Bayescan. Neutral data were used in two ways. First, to analyze neutral spatial genetic structure and correlate it with environmental variables (RDA) and compare with adaptive genetic structure. Second, to incorporate the neutral genetic signal in the detection of local adaptation candidate loci based on genotype-oceanographic associations.

**Table 1:** Sampling sites of local populations of the ascidian *Pyura chilensis* in the southeast Pacific. Name of site, site code, geographic coordinates and number of genotyped individuals (N).

Site	Code	Coordinates	N
Pan de Azúcar	PA	26°08′S; 70°39′W	30
Caleta Pajonales	CP	27°44′S; 71°02′W	29
La Herradura	LH	29°58′S; 71°21′W	30
Talcahuano	TH	36°38′S; 71°21′W	30
Ancud	AC	41°52′S; 73°50′W	30
Total			149

### Genomic-oceanographic associations

As expected, due to the way that outlier loci were detected, pairwise-*F*_ST_ matrix showed higher levels of genetic differentiation for outlier loci (putatively adaptive) than neutral loci (Table 2). Both *F*_ST_ matrices showed mostly significant values. For neutral loci, all pairwise differentiation values were highly significant. For outlier loci, *F*_ST_ values were mostly high and significant, with the exception of the comparison between the two northernmost sites, Pan de Azúcar (PA) and Caleta Pajonales (CP), which was non-significant (Table 2).

**Table 2:** Pairwise *F*_ST_ values for neutral (above diagonal) and outlier loci (below diagonal) in *Pyura chilensis* for the South east coast. Significant values after FDR correction are shown in bold.

Site	PA	CP	LH	TA	AC
PA	0	**0.006**	**0.021**	**0.058**	**0.065**
CP	0.010	0	**0.018**	**0.052**	**0.060**
LH	**0.252**	**0.273**	0	**0.068**	**0.074**
TA	**0.624**	**0.652**	**0.843**	0	**0.013**
AC	**0.663**	**0.699**	**0.869**	**0.018**	0

**Figure 3:**
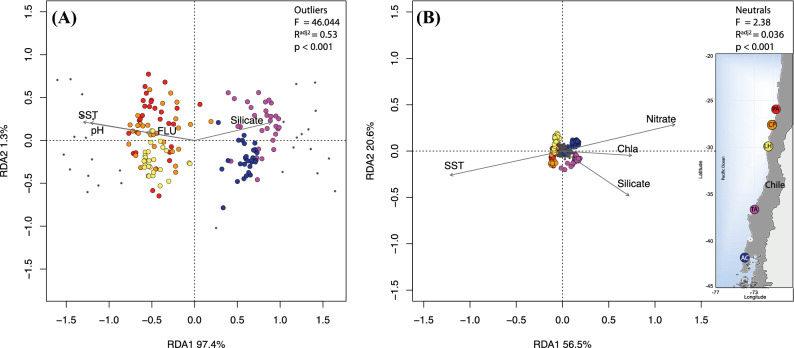
Redundancy analysis (RDA) showing the relative contributions of oceanographic variables to the genetic structure of outlier and neutral genotypes. SNP genotypes in gray; individuals are represented by different colors according to their location according to the map in the right panel. Plot shows the most relevant variables obtained with *ordistep* and *ordiR2step* functions.

With the information of the data obtained with outlier detection using population differentiation approaches (Bayescan, Out-flank, PCAdapt*)*, we determined the relative contribution of geographical position and environmental variables to the genetic structure of the outlier and neutral genotypes. For this, using the 29 consensus outlier loci, we carried on a multivariate correlation with environmental variables with a Partial Redundancy Analysis (Partial RDA’s). Optimal models were estimated incorporating oceanographic variables as fixed factors and controlling by spatial variables (dbMEMs), which resulted in a highly significant model (F = 46.04, Radj^2^ = 0.53, *p* < 0.001). The first two axes of the outlier loci model explained 98.4% of the total variance, and the distribution of the loci showed two groups, north and south of 30°S, that were significantly correlated to Sea Surface Temperature (SST), pH, fluorescence, and non-significant for silicate (Table 3, Fig. 3).

**Table 3:** Results of partial redundancy analysis (RDA) showing the relative contribution of each oceanographic and spatial variable (controlling the effect of space using vectors dbMEMs as co-variates) on the adaptive and neutral genetic structure of *P. chilensis*. Significant values associated for each variable are shown in bold. The acronyms of the environmental variables are the same as those used in Table S1.

Loci	Variable	Variance	F	*p* value	Radj^2^	*p* value
29 outliers	SST	0.01	4.65	**0.001**	0.534	**0.001**
	pH	0.01	6.23	**0.001**		
	FLU	0.004	1.86	**0.016**		
	Silicate	0.002	0.94	0.538		
	Residual	0.34				
2,521 Neutral	SST	0.002	4.99	**0.001**	0.036	**0.001**
	Nitrate	0.007	1.89	**0.001**		
	Silicate	0.006	1.59	**0.001**		
	Chl-a	0.004	1.05	0.193		
	Residuals	0.58

For neutral loci, the optimal general model was also highly significant (F = 2.38, *p* < 0.001), but with a much lower correlation value (Radj^2^ = 0.036). This model included SST, Nitrate, Silicate and Chl-a, being statistically significant only in the first three variables (Table 3, Fig. 3). The first two axes explained 77.1% of the total variance and the spatial distribution of the genotypes appear to be consistent with the geographical location of each sample site.

In summary, the RDA analyses showed dissimilar signals for the influence of environmental variables of putatively adaptive and neutral loci, except for the influence of SST. Although the neutral model was significant, the correlation coefficient was low, suggesting that neutral genotypes could be more associated with geographic distance than with environmental differences. Interpretation of these results was validated by the fact that a Partial Mantel Test using standardized matrices of genetic distance (*F*_ST_), geographic distance, and the *Mahalanovic environmental distance* (most relevant variables estimated with *ordistep* in RDA analysis) as a co-variate resulted in a highly positive correlation for neutral loci (r = 0.94, *p* = 0.008). Interestingly, the same model for outlier loci was not significant for geographic distance (r = 0.46, *p* = 0.08).

With neutral loci, we performed a Discriminant Analysis of Principal Components (DAPC) to estimate the number of genetic groups (*find.clusters* function) using the Bayesian Information Criterion (BIC), and to determine the neutral genetic structure. With this, DAPC identified an optimal *K* of two genetic groups; one north (PA, CP, and La Herradura (LH)) and another group south (Talcahuano (TH), Ancud (AC)) of 30° transition zone. Taking into the account that GEA analyses could be corrected with neutral structure, autocorrelation and geographic distance, this clustering was used in subsequent analyses to control for neutral effect in the determination of local adaptation candidate loci to avoid false positives.

In parallel to the implementation of outlier detection approaches based merely on genetic differentiation (e.g. *F*_ST_, ordination), to detect candidate loci for local adaptation we performed genotype environment association (GEA) analyses which correlate each environmental variable with individual genotypes. With the independent application of three different methods (LFMM, Bayenv2, SAMβADA), we detected a range of 315 – 666 putatively adaptive loci that exhibited significant correlation with each of the seven environmental variables. This result was substantially greater than those obtained with outlier detection approaches (Fig. 2B).

SST, pH and phosphate were the environmental variables with which most of the putatively adaptive loci were correlated (406, 402 and 378 loci, respectively), while Chl-a and silicate were correlated with fewer loci (235 and 132, respectively). A total of 94 local adaptation candidate lociwere identified by all three methods (LFMM, Bayenv2, SAMβADA), with 68% of the loci significantly associated with more than one environmental variable. In this context, the most relevant variables were pH (28), phosphate (26) and fluorescence (16), which detected most loci identified by the different methodologies, followed by SST (11) and Nitrate (6). In contrast, two and zero consensus loci, respectively, were detected for Chl-a and Silicate (Fig. 2B). Taking into the account all environmental variables and all the loci jointly identified as being under selection by the three methods, we identified a total 64 candidate loci for local adaptation.

**Figure 4:**
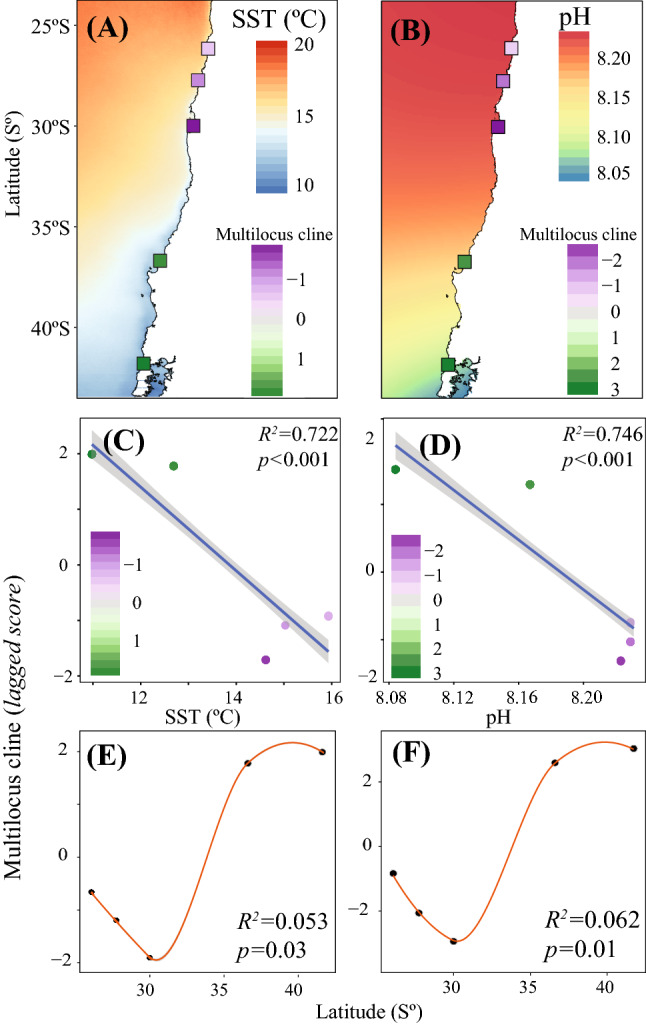
Spatial adaptive genetic variation of putatively adaptive loci for Surface Sea Temperature (SST) and pH in the 5 sites studied in *Pyura chilensis*. (**A**, **B**) Spatial structure of SST and pH throughout the study zone (Water color represents SST and pH variation) and values of the multilocus clines for each site measured by the *lagged scores* of the sPCA analysis, which reflect the genetic variability linked to the spatial structure between sites. (**C**, **D**) Scatter plot of linear adjustment between the multilocus clines determined in the *lagged scores* of the sPCA analysis for each location, considering the relationship between the oceanographic variables and the first axe of the lagged scores. The color gradient is the same used for panel (**A**, **B**). (**E**, **F**) shows dispersion graph of the multilocus clines determined in the sPCA analysis for each location considering the relationship between latitude and the first axe of the lagged scores using *loess smoothing* to fit the trend lines. Lineal models were carried on using the spatial vector associated with latitude (dbMEM2).

To asses if the variation in the candidate SNPs detected jointly by the GEA methods were significantly associated with each environmental variable, above the expected based only on the geographic proximity of sites, we used a spatial Principal Component analyses (sPCA). From sPCA’s, we extracted the *lagged scores* (multi-locus clines), which represent the genetic variability linked to the spatial structure among sites. The linear regressions performed with the multi-locus clines and the spatial variables (dbMEMs), indicated that the variables that best fit the adaptive genetic structure were SST (Radj^2^ = 0.722, *p* < 0.001, Fig. 4A–C) and pH (Radj^2^: 0.746, *p* < 0.001, Fig. 4B–D). The linear fit of these two variables to the adaptive genetic structure was in part due to the fact that the multi-locus clines associated with each site showed two geographic groups with clearly dissimilar *lagged scores* with small intra-group differences between sites (Fig. 4A,B); one north of 30°S (sites PA, CP and LH), and a southern group (TH and AC) (Fig. 4C–F). Such pattern was also recorded for the rest of the main environmental variables in which the associated regressions were significant, but with a relatively lower linear fit (fluorescence R^2 adjusted^ = 0.38, Phosphate R^2 adjusted^ = 0.65 and Nitrate R^2 adjusted^ = 0.59, see Figure S1). Interestingly, the spatial vector associated with latitude (dbMEM2) showed significant correlations with the multi-locus clines, but the adjusted R^2^ were near 0 and their fits on latitude were all nonlinear (Fig. 4E,F, Figure S2). These results indicated that putatively adaptive SNPs for each environmental variable exhibited stronger association with environmental variables than with spatial variables.

Both types of analysis, i.e. based on outlier detection (e.g. *F*_ST_, ordination) or based on genotype-environment association (GEA), were suitable and consistent for the search of relevant environmental factors in the adaptive structure of *P. chilensis* in the study area. The second type of methods (GEA), however, was more specific and allowed identification of a large number of putatively adaptive loci associated with each environmental variable independently.

### Genetic ontology

The 64 candidate loci for local adaptation identified previously with GEA methods were used to search for functional genes, as an exploratory analysis. Using restrictive filters to avoid false positives, the search revealed that five loci were significantly associated with annotated genes in the SWISS-PROT and GenBank databases. Three of these showed significant associations with up to three environmental variables (Table 4). The genes associated with these loci (Coiled-coil, Brachyury Protein, and Cyclin Dependent Kinase) have important functions in the biogenesis of cilia and in embryogenesis.

**Table 4:** Gene ontology of candidate loci to local adaptation in *Pyura chilensis*. Characterization of the matches obtained by BLAST analysis both in GenBank and in the Swiss-Prot databases for candidate loci. The locus, the number of associated environmental variables (N ENV), associated environmental variables (ENV), e-values, homology of the sequences (Ident.), taxonomic parameters (species, family, class) of the best match, the name of the gene/protein and its general biological function, if available, are specified. *SST genes identified only for Sea Surface Temperature (SST) at loci detected by at least two Genotype-environment association methods.

Locus	N ENV	ENV	UniProt ID	e-value	Ident	Species/family/class	Protein	Gen	Function
TP53299	4	pH phosphate, nitrate, SST	K7FSI5	1.00^E−08^	0.762	*Pelodiscus sinensis/*Trionychidae*/*Testudines	Coiled-coil and C2 domain containing 2A	CC2D2A	Biogenesis and cilium degradation
TP77542	3	pH, phosphate, fluorescence	H0YM91	2.00^E−04^	0.944	*Homo sapiens/*Hominidae*/*Mammalia	Brachyury protein	T	Transcription factor
TP2255	3	pH, phosphate, fluorescence	A0A1W2WCM2	7.00^E−04^	0.75	*Ciona savignyi/*Cionidae/Ascidiacea	cyclin-dependent kinase 5 activator 1-like	CDK5R1	Biological rhythms and development of central nervous system
TP73388	1	Chlorophyll a	Q9GR85	2.00^E−05^	0.857	*Halocynthia roretzi/*Pyuridae*/*Ascidiacea	HrTLC2 protein	RNA binding	Embryogenesis and body formation
TP57884	1	SST*	F7A786	5.00^E−04^	0.75	*Ciona intestinalis/*Cionidae /Ascidiacea	Phospholipid scramblase	PLSCR	Enzymatic phospholipid activity (scramblase)

**Figure 5:**
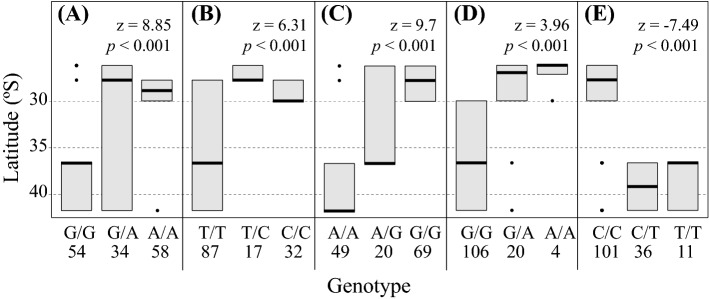
Genotype-latitude correlation in *Pyura chilensis* of 5 loci that showed significant correlation with environmental variables and matched with genes with known function in SWISS-PROSS. Boxplot displays the latitude of the sampling area and the three different genotypes of each of the 5 loci (**A**–**E**). In all the plots, the first genotype corresponds to the major allele in homozygous form. The order of loci is the same as Table 3 TP53299 correlated with pH, phosphate, nitrate, and SST; (**B**) TP77542 and (**C**) TP2255 correlated with pH, phosphate and fluorescence, (**D**) TP73388 correlated with chlorophyll-a, and (**E**) TP57884 correlated with SST.

For these five loci, all the General Lineal Models’ (GLM) showed significant differences in allele frequencies associated with latitude (Fig. 5A–E). The spatial structure showed that the homozygotes of both, the more frequent (major allele) (see Fig. 5B for the only exception) and the less frequent (minor allele) alleles, tended to be restricted north or south of 30°S, respectively (Fig. 5). Interestingly, in two of the five loci, both alleles (major and minor) were present in all the sampling area only as heterozygotes (Fig. 5A,C). In particular, the minor allele, with the exception of the TP57884 loci (Fig. 5E), was spatially restricted to north of 30°S in both, homozygosis and heterozygosis.

## Discussion

This study represents the first evaluation of the role of environmental factors in the adaptive processes of benthic species across a biogeographic transition zone (30°S) in the Humboldt Current System (HCS). A main conclusion is that the spatial genetic structure of neutral loci in *P. chilensis* was more influenced by geographic distance between sites than by environmental differences. Conversely, the genomic spatial structure of putatively adaptive loci was more influenced by environmental features. Specifically, adaptive genetic structure of *P. chilensis* across the HCS was better explained by the contrasting environmental conditions found north and south of the 30°S transition zone than by geographic distance.

The study of Segovia et al.^47^ was the first to suggest an active role of the environment in the maintenance of the 30° transition zone in the HCS at the genomic level. Likely, environmental differences north and south of 30°S, as reported by several studies^41,52–54^, could be shaping the genomic structure of the species. This transition zone has been considered as a historical biogeographical break^3^ (last maximum glacial period in the most recent estimations of separation for both regions), because only species with low capacity of larval dispersal have a coincident genetic differentiation at the 30°S (macroalgae^38,55^ and invertebrates^3,14,40,47^). In addition to the historic origin of the break, there are ongoing processes that are likely shaping adaptive genetic variation. Moreover, genetic differentiation of neutral markers does not reflect a genetic break at 30°S neither with mtDNA, nuclear traditional markers^8^ or neutral SNPs in *P. chilensis*^46^. This is consistent with several studies in were SNPs markers show finer genetic structure respect to traditional markers, but the general patterns tend to be in touch^56–59^. The fact that both, traditional markers (historical processes) and neutral SNPs (contemporary gene-flow) showed absence of genetic structure across the 30°S transition zone, suggests that anthropogenic transport is an effective agent of long-distance dispersal in the species^8,47^.

The main environmental factors that explain genetic structure in *P. chilensis* at 30°S in putatively adaptive loci were Sea Surface Temperature (SST), pH, upwelling-related variables (i.e., nutrients such as nitrate and phosphate), and photosynthetic activity measured by fluorescence (Flu), chlorophyll *a* (Chl-a). Association of these variables to genotypes tended to generate two consistent groups –north and south of 30°S – instead of multi-locus gradient clines. In spite of slight latitudinal variations, multi-locus clines showed an important intra-group (north and south of 30°S) degree of cohesion and accordingly, the most plausible scenario is the presence of two independent groups. Environmental differences north and south of the 30°S transition zone^41^ may constitute key factors in maintaining the genetic differentiation in spite of transport of individuals between local populations^8,47^. In the context of our study model, natural selection seems to override the homogenizing effect of gene flow across the 30°S transition zone of the HCS in putatively selected loci and accordingly lead to local adaptation processes.

In addition to the commonly reported effects of temperature on the genetic structure, the relevance of environmental variables associated with upwelling in maintaining adaptive genetic differentiation across the transition zone of the HCS in *P. chilensis* could be due to the presence of two areas with contrasting differences in terms of upwelling intensity. The first one, between 30°S and 18°S in northern Chile, includes upwelling-favorable winds across the year. The second one, between 30°S and 41°S, exhibits a marked seasonality in upwelling, especially in the austral spring and summer^52,60^, which also produces cold water near the coast from 30° southward^52^. The influence of environmental variables associated with temperature and productivity on the ecological structuring in near-shore marine benthic organisms has been widely reported both in the northeast and southeast Pacific coasts^61–63^. These studies suggest that upwelling regimes have an influence on the communities in terms of recruitment rates, biomass, and individual growth of the organisms^64^. More specifically, the concentration of ecological suppliers of primary productivity (i.e. nutrients) tend to show a variation in their growth rate along with upwelling and that the greatest response occurs when upwelling is intermittent^64,65^.

For the particular case of *P. chilensis*, eco-physiological differences have been reported across the study area^66–68^ which together with the limited capacity of the species to colonize new areas, despite being a dominant substrate competitor^50,69^, suggest an active role of local adaptation. The weak colonizing capacity of *P. chilensis* may be a consequence of non-selective larval settlement that could be influenced by water flow, coastal configurations, ecological interactions^45^, and other selective environmental variables. For example, differences in SST have a significant influence on the seasonal and spatial reproductive activity of *P. chilensis*^43^*.* Despite this, it has also been reported that the species may have a wide range of physiological tolerance to temperature^70^, which could explain its wide geographic distribution range (10°–44°S)^42^. It is possible that the wide physiological tolerance range may be counteracted by adaptive genetic differentiation along the species distribution. Local adaptation could lead to genotypic differences between adjacent populations inhabiting contrasting environmental conditions. In fact, recently Giles et al.^71^ and Morales-González et al.^72^ showed that *P. chilensis* is significantly structured over fine spatial scales (20–40 km). Consistent with this finding, the genetic diversity of candidate loci for local adaptation varied along the study area. Both, major and minor alleles, were in some cases present as heterozygotes along the study area but, particularly the minor allele, tended to be geographically restricted to the region north of 30°S. This suggests that transport of individuals between regions by anthropogenic vectors could allow the recruitment of individuals in areas with sub-optimal conditions for genetic migrants, as proposed by Haye & Muñoz-Herrera^8^ and Segovia et al.^47^.

From all candidate SNPs, several potential genes with known function were significantly associated with one or more environmental variables. However, since GBS loci are random short loci distributed across the genome, gene mapping could be highly influenced by chance, or by hitchhiking^73–75^. Taking this caveat into account, one locus was consistently and significantly correlated with four environmental variables (SST, pH, nitrate, phosphate) and its sequence matched that of a gene that appears to be involved in cilia-mediated processes, including filtration and ventilation. Interestingly, temperature and phytoplankton concentration appear to be important environmental variables that control fundamental processes associated with ciliate-mediated filtration in ascidians^76,77^. A positive relationship between filtration rate (cilia activity) and temperature has been described in the ascidian *Ciona intestinalis*^78^. Cilia activity also varies with water productivity, with a negative relationship between cilia activity and phytoplankton biomass^78^. Thus, both changes in productivity and temperature could have a significant effect on the rate of cilia mediated filtration and ventilation in *P. chilensis,* which could be a key factor in the maintenance of contemporary genetic structure and restriction of certain alleles north and south of 30°S.

Finally, in addition to the role of upwelling, the influence of the pH, nutrients such as phosphate and photosynthetic activity in the genomic structure of *P. chilensis* may indicate the influence of continental waters, especially river discharges^79^. The influence of continental waters is also variable in the study zone; river discharges have little influence north of 32°S while between 36°S and 42°S there is a considerable influence of continental waters, particularly during autumn and winter^80^. The multilocus clines associated with putatively adaptive loci showed similar values in the two sites south of 30°S, which suggests that the allele frequencies varied less than expected on the basis on considerable distance between them (~ 580 km). Such genomic homogeneity in the putatively adaptive genetic structure south of 30°S may be due to the influence of continental waters (glaciers and river discharges) occurring mainly in that area^79^ combined with the effect of anthropogenic transport that allows dispersion of individuals^47^.

## Conclusions

Signatures of selection and local adaptation can now be evaluated in populations across entire genomes or genome sampling using population differentiation approaches (i.e. outliers) or in association with environmental variables to test the influence of biotic and abiotic factors in the spatial genomic structure. These evaluations give insights into contemporary processes, and may explain how environmental factors influence selective and neutral genomic diversity within and among populations^19^. Remote sensing of oceanographic variables can be quantified across relevant spatial scales, and including genomic diversity analyses, allows to better understand how seascape attributes can shape the spatial genetic structure in near-shore marine organisms^20,37,81^. This study represents the first attempt to evaluate the association between environmental factors and the maintenance of a genetic discontinuity across the HCS in a species for which previous studies showed genetic homogeneity at a large spatial scale, suggesting active gene flow along the southeastern Pacific coast. The main results suggest that at the recorded transition zone (30°S), local adaptation processes could allow the maintenance of contemporary genomic differentiation and spatial genetic structure in *P. chilensis*. This species is an example of how seascape heterogeneity across a biogeographic transition zone may override the effects of gene flow and lead to local adaptative processes. Even in the presence of high gene flow, probably mediated by anthropogenic transport between and within regions, *P. chilensis* appears to be sensitive to environmental heterogeneity, especially for those variables that could influence fundamental processes like feeding, filtration, recruitment and growth. Accordingly, putatively adaptive loci showed strong genetic structure associated mainly with environmental differences north and south of 30°S biogeographic transition zone.

The environmental variables used in this study may be a first step in identifying the selective factors that maintain the current genetic structure north and south of 30°S in other benthic marine species from the southeastern Pacific coast.

## Methods

### Genomic data collection

Raw data from Segovia et al.^47^ were re-analyzed adding 89 individuals obtained from the same sampling sites, the year after the original sampling (2014–2015). From the original dataset, to avoid bias in the candidate loci detection, the location of Los Molinos (~ 39°S) was excluded due to the presence of a highly differentiated lineage^47^. In total, thirty individuals of *Pyura chilensis* were collected from each of five localities between 26°S and 42°S from two consecutive years (2014–2015) in the Southeast Pacific Coast (Fig. 1; Table 1). To avoid kinship bias, individuals were sampled at least 2 m apart^72^. DNA was extracted from mantle tissue using the DNeasy Blood & Tissue kit (QIAGEN) following the manufacturer’s instructions. The DNA was sequenced using the restriction site-associated DNA sequencing method Genotyping-by-Sequencing^82^ using the *ApeKI* restriction enzyme. This enzyme was used following the optimization of Segovia et al.^47^ for the species, which was chosen due to the wide genome distribution of flanking region and avoiding of repetitive zones^83^. Libraries were prepared in a HiSeq2000 (Illumina, USA) platform and the resulted reads (100pb) were visualized and analyzed in FastQC version 0.10.1 for quality checks. Demultiplexing and SNP calling filters were done following Segovia et al.^47^ using the Universal Network-Enabled Analysis Kit pipeline (UNEAK^84^) from the TASSEL platform (www.maizegenetics.net) using a minimum call rate (mnC) of 85% and a minor allele frequency (mAF) of 0.04 excluding also those loci in Hardy–Weinberg disequilibrium in at least 60% of the sampled sites after a False Discovery Rate (FDR) with a q-value of 0.05.

### Genomic-oceanographic associations

We analyzed the effect of environmental factors on the genetic structure of *P. chilensis* using seven relevant oceanographic variables for each locality (Table S1). Environmental data were obtained from the remotely sensing database of Aqua-MODIS and SeaWiFS from NASA (Level-3, 4 km^2^, 8-day composite images; https://oceancolor.gsfc.nasa.gov) and from the Bio-Oracle database (Ocean Rasters for Analyses of Climate and Environment, 5 Arcmin [9.2 km]^85,86^; see Table S1). We included in the analyses two types of candidate loci determination based on 1) outlier loci detection (*F*_ST_ and ordination based), and 2) Genotype-environmental (oceanographic) association analysis.

For outlier detection, three independent methods were used: 1) Bayescan 2.177^87^ analyses were performed with a *pr_odds* of 1,000 with 100,000 iterations and a burn-in of 10,000 steps. Results were corrected using a FDR of 0.05 with the logarithm of the *q values*. The candidate loci for positive selection considered were those with strong or very strong evidence of selection according to Jeffrey’s criterion^88^ based on the values of the Bayes Factor (bf > 10).

2) Out-flank^89^ analyses were performed using a neutral *F*_ST_ distribution, eliminating loci with extreme *F*_ST_ values in both tails, and using a LeftTrimFraction and RightTrimFraction of 0.05, with a minimum heterozygosity of 0.1. The outliers were identified in the 5 sampling sites using an interval of q values of 0.05. Finally, 3) PCAdapt^90^ were done by first using a PCA with the number of groups (K) equal to the number of populations studied (5) to define the optimal value of K. Distribution of *p* values was corrected using a FDR with q-values of 0.05, finally obtaining a list of outlier loci that were candidates for selected loci (See Appendix 2 and Table S2 for detailed methods). Multiple approximations were used to reduce the probability of false positives in the final dataset following Lotterhos & Whitlock^91^ and Rellstab et al.^18^.

Using the information of the data obtained with outlier detection using population differentiation approaches (Bayescan, Out-flank, PCAdapt), we determined the relative contribution of geographical position and each considered environmental variable to the genetic structure of outlier and neutral genotypes.

### Spatial structure and environmental association analyses

The spatial genomic structure of *P. chilensis* was estimated with the geographic coordinates of the sampling sites using distance vectors based on distance-based Moran’s eigenvector maps, dbMEMs^92,93^. Following this, dbMEMs were analyzed in Redundancy Analyses (RDA) in the R package vegan^94^ using standardized environmental variables and the genotypes associated with the neutral and positive selection candidate loci as response variables. Prior to the analysis, genotype data were standardized by removing the broad scale trend using the *decostand* function with the Hellinger’s method in vegan. With this, we carried out a Partial RDA controlling for the effect of space (dbMEMs). Optimal model in vegan were determined using *ordistep* and *ordiR2step* functions and the significances were evaluated for each fixed factor through a marginal ANOVA test (10,000 permutations). Finally, we calculated the pairwise *F*_ST_ matrices for both datasets using Arlequin 3.5.2.2^95^ using 10,000 permutations. With this, we estimated the relation between genetic, geographic and environmental distances using standardized values with a Partial Mantel test in *ecodist* package in R. This test was carried on using genetic distance (i.e. *F*_ST_) and geographical distance between localities, using an environmental distance matrix as covariate. The environmental distance matrix was calculated using *Mahalanovic distances* of the standardized oceanographic variables (SST, Chl-a, Fluorescence, pH, Nitrate, Phosphate and Silicate) in the R package *StatMatch*. Final matrix was constructed with the *vegdist* function of vegan in R, using a Euclidean method.

In parallel, we also carried out GEA approaches, that explicitly incorporated the environmental variables to determine candidate loci for local adaptation. In order to avoid false positives due to neutral processes and/or spatial autocorrelation^18^ we used both, multiple approximations and the signal of previously determined neutral loci as control^18,91,96,97^. For this, the *find.clusters* function of a Discriminant Analysis of Principal Components (DAPC) of putatively neutral SNPs was performed first in adegenet^98^ package of R 3.22. This was done to determine the number of genetic groups/clusters using neutral loci with a Bayesian Information Criteria (BIC). Once the neutral structure was determined, we used three approximations: (1) LFMM v 1.4 (Latent Factors of Mixed Models)^99^, which were executed in the LEA package in R 3.22 using the number of latent factors based on the results of the DAPC (optimum K of the neutral structure). 200,000 iterations were done after a burn-in of the first 20,000 steps. Five independent runs were done for each analysis to estimate the different parameters, as recommended by Frichot et al^99^, and the mean of the z-scores was used for the final results. The significance of the values associated with each SNP was calculated using α = 0.01 corrected with a FDR.

(2) With Bayenv2^100^ we evaluate the correlation between SNPs and environmental factors testing whether the model that includes the environmental variable (each variable is evaluated separately) fits the data better than a null model (pairwise covariance differentiation matrix with neutral SNPs). To estimate this matrix, we performed five independent runs using 100,000 iterations. Then the entire set of SNPs was tested individually with the environmental standardized variables using 80,000 MCMC iterations. To identify candidate SNPs with evidence of positive selection for each environmental variable we used Bayes factor > 10 according to Jeffrey’s criterion^88^.

Finally, with (3) SAMβADA^101^ we tested for Genotype-Environment associations considering neutral structure based both, on the number of groups present (optimum K determined by DAPC) and the spatial autocorrelation using the information of the geographic coordinates of the studied sites. We used a multiple logistic regression in which the *p* values were calculated from regressions of all possible genetic-environmental associations according to a *Wald Score Test*. The results were compared to a χ^2^ distribution with one degree of freedom. The loci with q-values less than 0.05, corrected with an FDR, were considered to have significant associations with a specific environmental variable.

Once all consensus candidate SNP loci for local adaptation were obtained, i.e. for each environmental variable and each of the three methods (LFMM, Bayenv2, SAMβADA), spatial structure of loci was analyzed independently using spatial Principal Components Analyses (sPCA), in adegenet. These analyses were performed to infer if the variation in the candidate SNPs was significantly associated with each environmental variable above that expected based only on the geographic proximity of sites. For this, we extracted the *lagged scores* associated with the first two principal components for each site. The *lagged scores* were then used to transform the genetic variation of the candidate SNPs into multilocus geographic clines. To evaluate if the variation of the candidate SNPs correlated best to environmental or spatial variables, the multi-locus clines were used to perform linear regressions with both groups of variables using dbMEM vectors. For statistical reasons, only those sets including more than five loci were retained.

### Genetic ontology

As an exploratory analysis, *tags* of detected putatively adaptive loci for each environmental variable were extracted to identify potential genes with known functions available in the SWISS-PROT database^102^ and in GenBank (https://www.ncbi.nlm.nih.gov/genbank/). In the case of Sea Surface Temperature (SST), we used tag sequences of those loci that were significantly correlated in at least two of the three genotype-oceanographic association approaches. SNPs were filtered by e-values preserving only matches greater than 1 × 10^−4^ and a minimum of 75% homology. The tag sequences associated with candidate loci were used as query sequences for BLAST searches executed in the database of annotated genes from SWISS-PROT.

Using positive matches for Gene Ontology, we used a General Lineal Model (GLM) with binomial error to correlate the genotypes of each candidate locus SNP and the latitude using R environment.

## Data availability

Raw SNP-GBS data (.vcf file) for each sample and environmental variable were deposited in the Bitbucket database: https://bitbucket.org/NicoSegoviaC/seascape_pyurachilensis/src/master/.

## Supplementary information

Supplementary file1
