# Supplementary material for: Signatures of local adaptation in the spatial genetic structure of the ascidian Pyura chilensis along the southeast Pacific coast
Source: Sci Rep. 2020 Aug 24;10:14098. doi: 10.1038/s41598-020-70798-1 (PMC7445245; doi:10.1038/s41598-020-70798-1)
Supplement: Supplementary file 1 — Supplementary file1 [file 41598_2020_70798_MOESM1_ESM.docx]

Supplementary Material

For

**Signatures of local adaptation in the spatial genetic structure of the ascidian *Pyura chilensis* along the southeast Pacific coast**

**NICOLÁS I. SEGOVIA^1,2^, CLAUDIO A. GONZÁLEZ-WEVAR^2,3,4^, PILAR A. HAYE^1^***

1. Laboratorio de Diversidad Molecular, Departamento de Biología Marina, Facultad de Ciencias del Mar, Universidad Católica del Norte, Coquimbo, Chile.
2. Instituto de Ecología y Biodiversidad IEB, Departamento de Ciencias Ecológicas, Facultad de Ciencias, Universidad de Chile. Las Palmeras 3425, Ñuñoa, Santiago, Chile.
3. Instituto de Ciencias Marinas y Limnológicas (ICML), Facultad de Ciencias, Universidad Austral de Chile, Casilla 567, Valdivia, Chile.
4. Centro FONDAP de Investigaciones en Dinámica de Ecosistemas de Altas Latitudes (IDEAL). Universidad Austral de Chile, Casilla 567, Valdivia, Chile.

***Corresponding author:** Pilar A. Haye, E-mail: [**phaye@ucn.cl**](mailto:phaye@ucn.cl)

### **Appendix 1**

### **Oceanographic data collection**

Table S1: Oceanographic variables included in the analysis of seascape genomics of *Pyura chilensis*.

The environmental variables (with its acronym in parentheses), the source of data of origin, the temporal and spatial resolution (if available), the date of beginning and end of the range of data considered and the unit of measurement of the variable studied. For the data obtained from Aqua-MODIS, a 10-year interval was chosen up to the sampling date (2005-2015). For the data set extracted from Bio-Oracle, the time interval is previously determined.

| Variable | Source | Tem.Res | Sp. Res. | Beg. date | End date | Unit |
| --- | --- | --- | --- | --- | --- | --- |
| Sea Surface Temperature-Night Only (SST) | Aqua-MODIS | Monthly | 4 km | 2005 | 2015 | C |
| Chlorophyll a concentration (Chl-a) | Aqua-MODIS | Monthly | 4 km | 2005 | 2015 | mg m-3 |
| Normalized fluorescence line height (Flu) | Aqua-MODIS | Monthly | 4 km | 2005 | 2015 | mW cm^-2 um^-1 sr^-1 |
| Sea Surface Temperature (SST) /Control | BIo-Oracle/Aqua-MODIS | Monthly | 5 arcmin (9.2km) | 2002 | 2009 | ºC |
| Nitrate | Bio-Oracle/World Ocean Data BASE | - | In Situ measured | 1928 | 2008 | umol/l |
| pH | Bio-Oracle/World Ocean Data BASE | - | In Situ measured | 1910 | 2007 | Unitless |
| Phosphate | Bio-Oracle/World Ocean Data BASE | - | In Situ measured | 1910 | 1986 | umol/l |
| Silicate | Bio-Oracle/World Ocean Data BASE | - | In Situ measured | 1930 | 2008 | umol/l |

**Appendix 2 Detailed Seascape genomics methods.**

***Population differentiation analysis and Redundancy analysis methods***

The spatial structure was estimated with the geographic coordinates of the sampling sites using distance vectors based on distance-based Moran’s eigenvector maps, dbMEMs (Dray et al. 2006, Legendre and Legendre 2012). The dbMEMs were determined by converting latitude and longitude to Cartesian coordinates using the *SoDa* package in R 3.22. Then, a matrix of Euclidean distances between these cartesian coordinates were calculated (using the *dist* function in VEGAN), and finally the dbMEMs were estimated using the create.dbMEM.model function in the ADESPATIAL package. This created a spatial matrix with two vectors, one associated with longitude (dbMEM1) and the other with latitude (dbMEM2), which were then used in redundancy analyses (RDA).

The RDAs, were performed in VEGAN (Oksanen et al. 2017) using the dbMEMs, environmental variables, and the genotypes associated with the neutral and candidates’ loci for positive selection detected by the three PDA methods as response variable. Before the analyses, genotype data were standardized by removing the broad scale trend using the *decostand* function with the Hellinger’s method in VEGAN (Oksanen et al. 2017). We executed three RDA models; 1) A full RDA (dbMEMs and Enviromental variables as fixed factors), 2) a “spatial RDA” that used the environmental variables as fixed factors and dbMEM vectors as co-variables, to control the effect of the spatial distribution in the genetic structure, and 3) an “environmental RDA” with the dbMEM vectors as fixed factors and the environmental factors as conditional variables (co-variables). For the three kinds of RDA, we determined the optimal model with respect to the environmental and/or spatial factors that best explained the genetic variability using the *ordistep* function in VEGAN. This function selects the variables that constitute the optimum model according to their significance, R^2^ adjusted value (*or2distep* function), and the value of the Akaike Information Criterion (AIC). For each model, we used a marginal ANOVA with 10,000 permutations to evaluate the significance of each fixed factor considered.

We also performed EAA that explicitly incorporate the environmental variables in the determination of candidate loci for local adaptation. To identify candidate loci for local adaptation and to avoid false positives in the EAA we used both multiple approximations and the signal of neutral loci as control (De Mita et al. 2013, de Villemereuil et al. 2014, Frichot et al. 2015, Lotterhos and Whitlock 2015, Rellstab et al. 2015). For this, first a Discriminant Analysis of Principal Components (DAPC) of putatively neutral SNPs were performed in ADEGENET (Jombart 2008) package of R 3.22. In DAPC, we used the k-means and a Bayesian Information Criterion (BIC) to identify the optimal number of clusters in the data. The number of clusters in each data set was determined using 10e^7^ iterations. To avoid unstable assignments in each cluster, 50 PCs were retained (N_total_/3), using all discriminant functions.

Once the neutral structure was determined, we used three different approximations to search for environment-SNP associations: 1) LFMM v 1.4 (Frichot et al. 2013), 2) BAYENV2 (Guenther and Coop 2013) and 3) SAMβADA (Stucki et al. 2016) (Supporting Information, Table S2).

Once the common candidate SNP loci for local adaptation were detected through BAYENV2, LFMM and SAMβADA for each environmental variable, they were extracted from the global dataset and analyzed independently using spatial principal components analyses (sPCA), in the ADEGENET package (Jombart 2008) in R 3.22. sPCA is a multivariate analysis that searches for discrete or clinal genetic groups based on the allele frequencies of the candidate loci (Jombart 2008). This approximation includes both spatial information based on the geographic coordinates and genetic data. We used sPCA to infer if the variation in the candidate SNPs is significantly associated with each environmental variable above what would be expected based only on the geographic proximity of each site. To execute the sPCA we used a geographic (spatial) proximity network among localities through the *neighborhood-by-distance* method, using the geographic coordinates of each studied site (Joombart 2008). From this, we extracted the *lagged scores* associated with the first two principal components for each locality, which reflects the genetic variability that is linked to the spatial structure among sites. The *lagged scores* were used to transform the genetic variation of the candidate SNPs into multilocus geographic clines. To evaluate if the variation of the candidate SNPs correlate best to environmental or the spatial variables, the multi-locus clines were used to perform linear regressions with both groups of variables using dbMEM vectors.

Three independent approximations based on PDA were used to detect loci putatively subject to positive selection: 1) BayeScan 2.1 implements a Bayesian method based on a logistic regression model that separates loci putatively affected by selection (locus-specific putatively adaptive genetic variation) from those affected by population-specific demographic processes (null model or neutral genetic variation). This analysis detects signals neutral to selection and both positive and balancing selection signals. Those under balancing selection were eliminated from the dataset for later analyses; since these loci have lower differentiation values than that expected for neutral variation they may hid the neutral structure. We used an *a priori* model (pr_odds parameter) with 100,000 iterations and a burn-in of 10,000 steps. The results were corrected using a false discovery rate of 0.05 with the logarithm of the *q* values. The candidate loci for positive selection considered were those with strong or very strong evidence of selection according to Jeffrey’s criterion (Jeffrey 1961) based on the values of the Bayes Factor (*bf* > 10).

The second PDA method used, 2) OUT-Flank, estimates a probability based on a distribution fitted to the values of F_ST_ to infer the distribution of F_ST_ for putatively neutral loci. This distribution is used to assign *q* values to each locus and detect loci with outlier F_ST_ values. To do this we eliminated loci with extreme F_ST_ values in both tails, using a LeftTrimFraction and RightTrimFraction with 0.05 in both cases, as well as a minimum heterozygosity of 0.1. The outliers were identified in the 5 sampling sites using an interval of *q* values of 0.05.

Finally, 3) PCAdapt uses a principal component analysis (PCA) to determine the percentage of genetic variance explained by each component. This was done by first using a PCA with the number of groups (K) equal to the number of populations studied (5). Based on this result, we then defined the optimal value of K based on the highest K value after stabilization of the curve of the proportion of variance explained as a function of the number of principal components stabilized (Luu et al 2017). For a given SNP, PCAdapt uses a test of Mahalanobis distance to determine if a marker is or is not an outlier based on the factor loadings, which are the correlation between this SNP and each principal component. The Manhattan graphs to define the outliers were made using log_10_ *p* values. The distribution of *p* values was corrected using a false discovery rate of 0.05, finally obtaining a list of the outlier loci that are candidates for selected loci.

***Environmental Association Analysis***

Once the neutral structure was determined, to look for environment-locus SNP associations we used three EAA approximations (Figure 1) implemented in: 1) LFMM v.1.4) (Frichot et al 2013), 2) Bayenv2 (Guenther & Coop 2013) and 3) Samβada (Stucki et al 2016). The LFMM (Latent Factors of Mixed Models) analysis determines associations between environmental variables and SNPs using environmental variables as fixed effects and neutral genetic structure as a latent or unobserved factor (Frichot et al 2013). The program was executed using the number of latent factors based on the results of the DAPC (optimum K of the neutral structure) using 200,000 iterations after a burn-in of 10,000. Five independent runs were done for each analysis to estimate the different parameters, as recommended by Frichot et al (2013), and the mean of the z-scores was used for the final results. All these were executed in the LEA package of R 3.22 and the significance of the values associated with each SNP was calculated using α = 0.01 corrected with a False Discovery Rate.

The second EAA approximation used was Bayenv2 (Guenther & Coop 2013). This program uses a Bayesian framework and evaluates correlations between molecular markers and environmental factors, testing whether the model that includes the environmental variable (each variable is evaluated separately) fits the data better than a null model. The null model is a genetic covariance matrix analogous to a F_ST_ matrix between pairs of populations, calculated using the estimated allele frequencies of neutral loci. To estimate this matrix, we performed five independent runs using 100,000 iterations to avoid bias due to the number of MCMA iterations, and once matrices were obtained that were not significantly different, we generated a mean matrix using the last iteration of each of the 5 runs (Frichot et al 2013). Then the complete set of SNPs was tested individually with the 10 environmental variables previously standardized (mean=0, variance =1) using 80,000 MCMC iterations with a sampling interval of 500 iterations. To identify SNP candidates with evidence of positive selection for each environmental variable we used Bayes factor >10 (Kass & Raftery 1995), according to Jeffrey’s criterion (Jeffreys 1961).

Finally, we used the Samβada program (Stucki et al 2016) to test for associations between allele frequencies and environmental parameters among sites, considering the neutral structure based both on the number of groups present (optimum K determined by DAPC) and the spatial autocorrelation using the information of the geographic coordinates of the studied sites. Samβada implements a univariate analysis of multiple logistic regressions in which the p values are calculated from regressions of all possible genetic-environmental associations according to an analysis called the Wald Score Test. The results are compared to a Chi squared distribution with one degree of freedom. The loci with p values less than 0.05, corrected with an FDR, were considered as significant associations between the corresponding marker and environmental variable

Table S2: Population Differentiation Analysis and Environmental-Association Analysis for seascape genomics in *Pyura chilensis.*

Table shows the details of each method used for the determination of diversifying selection candidates in *Pyura chilensis*.

| Method | Software | Detail | Citation |
| --- | --- | --- | --- |
| Population differentiation analysis (PDA) | BAYESCAN 2.1 | Bayesian method based on a logistic regression model that separates loci putatively affected by selection (locus-specific putatively adaptive genetic variation) from those affected by population-specific demographic processes (null model or neutral genetic variation). | Foll & Gaggiotti, 2008 |
|  | OUT-FLANK | Estimates a probability based on a distribution fitted to the values of F_ST_ to infer the distribution of F_ST_ for putatively neutral loci. | Whitlock & Lotterhos, 2015 |
|  | PCADAPT | Uses a principal component analysis (PCA) to determine the percentage of genetic variance explained by the first two components and determinate which loci are explained most the observed genetic structure and thus candidates to positive selection. | Luu et al., 2017 |
| Environmental-Association Analyses (EAA) | LFMM v 1.4 | Determines associations between environment and SNPs using environmental variables as fixed effects and neutral genetic structure as a latent or unobserved factor | Frichot et al., 2013 |
|  | BAYENV2 | Uses a Bayesian framework to testing whether a model that includes the environmental variable (each variable is evaluated separately) fits the data better than a null model. This null model was represented by a genetic covariance matrix analogous to a F_ST_ matrix between pairs of populations, calculated using the estimated allele frequencies of neutral loci. | Guenther & Coop, 2013 |
|  | SAMβADA | test for associations between allele frequencies and environmental parameters among sites, considering the neutral structure based both on the number of genetic groups (optimum K determined by DAPC) and the spatial autocorrelation using the information of the geographic coordinates of the studied sites. | Stucki et al., 2016 |

**Appendix 3** Spatial genetic structure correlated with environmental variables.


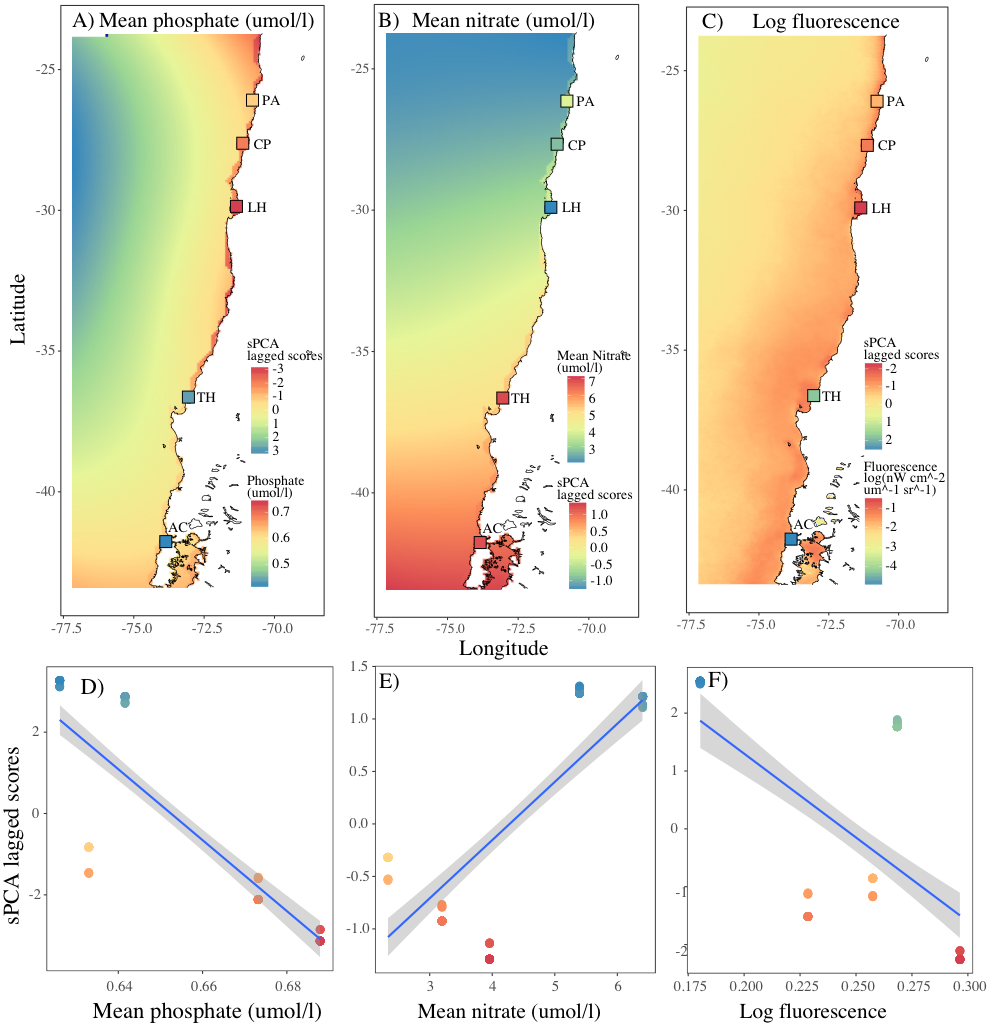


Figure S1: Spatial structure of phosphate, nitrate and fluorescence throughout the study zone (Water color represents their respective variations) and values ​​of the multilocus clines for each site. Multilocus clines were measured by the *lagged scores* of the sPCA analysis, which reflect the genetic variability which is linked to the spatial structure between sites.


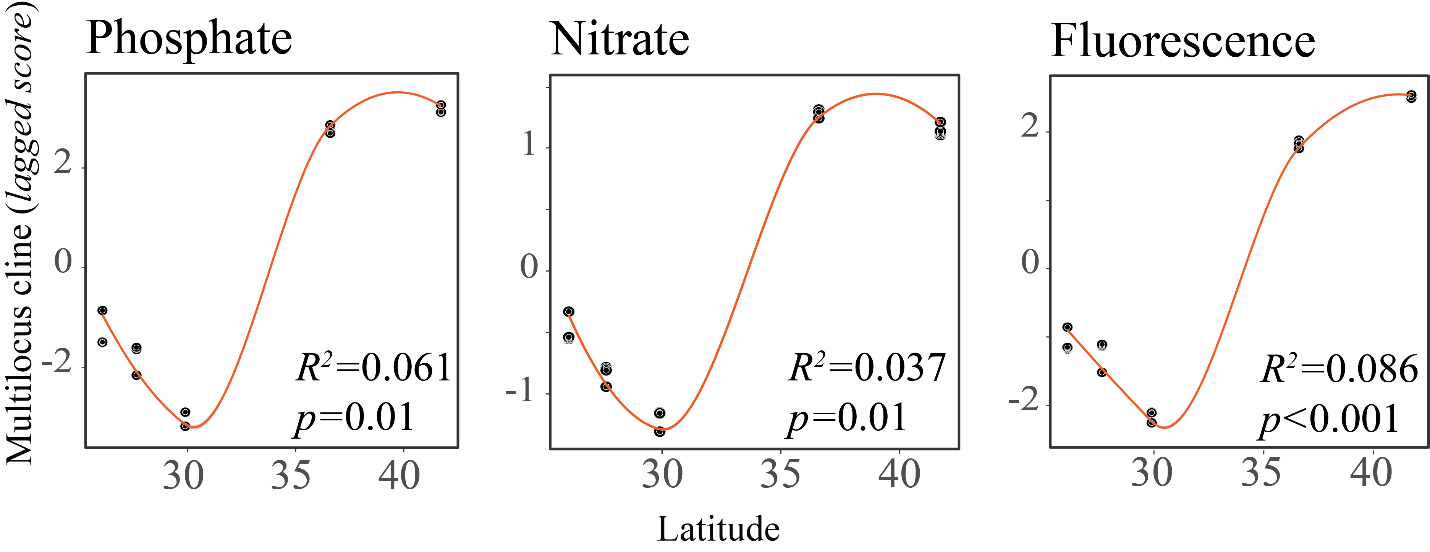


Figure S2: Spatial structure of phosphate, nitrate and fluorescence throughout the study zone and values ​​of the multilocus clines for each site. Multilocus clines were measured by the *lagged scores* of the sPCA analysis, which reflect the genetic variability which is linked to the spatial structure between sites. Figures shows the dispersion of the multilocus clines determined in the sPCA analysis for each location considering the relationship between latitude and the first axe of the lagged scores using *loess smoothing* to fit the trend lines. Lineal models were carried on using the spatial vector associated with latitude (dbMEM2).
